# Supplementary material for: The interactome of intact mitochondria by cross-linking mass spectrometry provides evidence for coexisting respiratory supercomplexes
Source: Mol Cell Proteomics. 2017 Dec 8;17(2):216–32. doi: 10.1074/mcp.RA117.000470 (PMC5795388; doi:10.1074/mcp.RA117.000470)
Supplement: Supplemental Data [file supp_RA117.000470_133922_0_supp_23978_fzffwf.zip › spectra_annotation/mito_spectra_annotation/389-1-3-1-2-1.pdf]

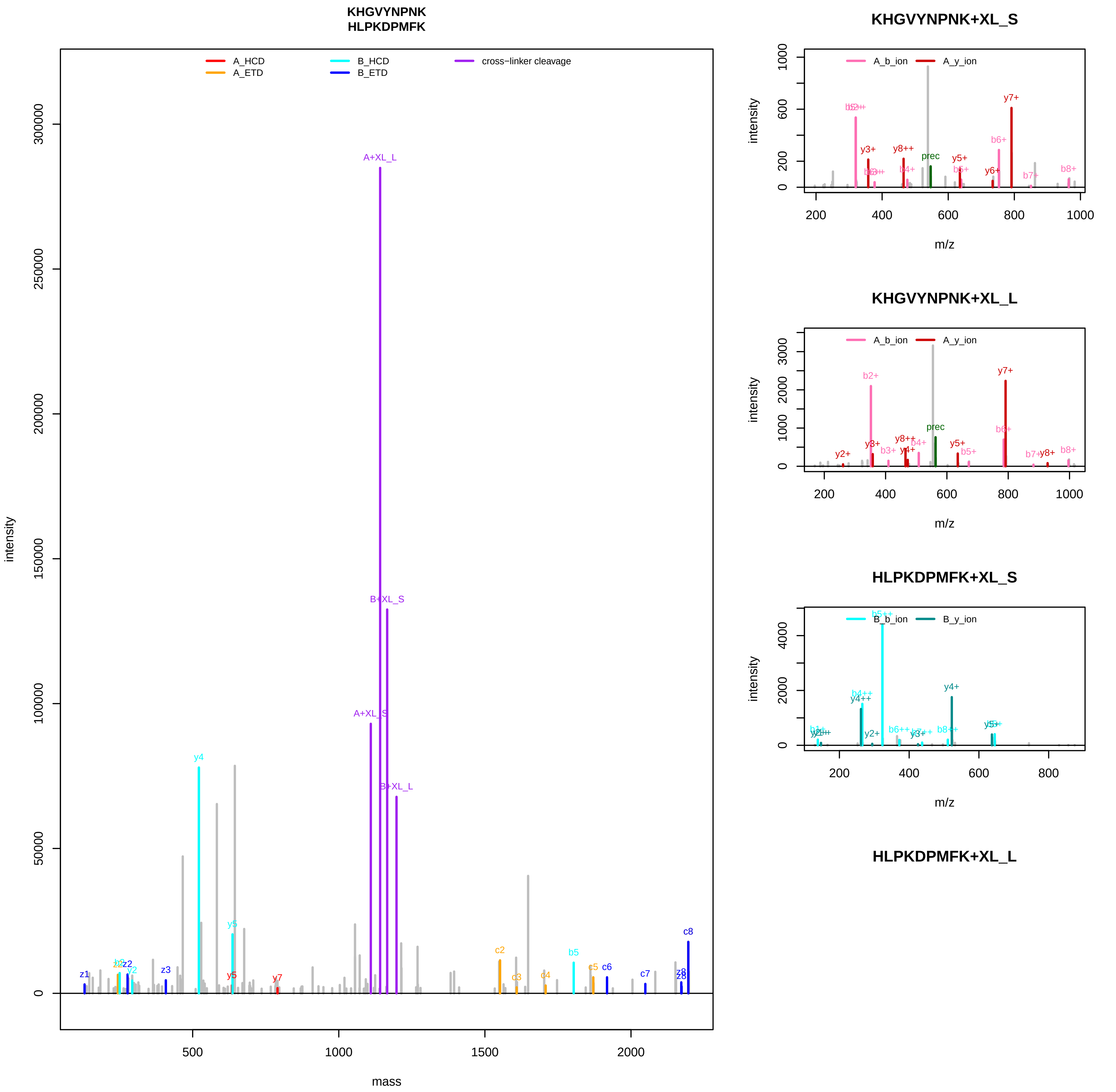
KHGVYNPNK
HLPKDPMFK
A_HCD
A_ETD
B_HCD
B_ETD
cross−linker cleavage
A+XL_L
B+XL_S
A+XL_S
B+XL_L
y4
y5
y7
z1
z2
z3
b2
y2
c2
c3
c4
c5
c6
c7
c8
b5
z8
intensity
mass
0
50000
100000
150000
200000
250000
300000
500
1000
1500
2000
KHGVYNPNK+XL_S
A_b_ion
A_y_ion
y7+
y8++
y3+
y5+
y6+
b6+
b7+
b8+
b4+
prec
m/z
KHGVYNPNK+XL_L
b2+
y2+
y3+
b3+
y4+
b4+
y5+
b5+
b6+
b7+
y8+
HLPKDPMFK+XL_S
B_b_ion
B_y_ion
b5++
b4++
y4++
y4+
y2+
y3+
b6++
b8++
y5+
HLPKDPMFK+XL_L
